# Removal of a frozen elephant trunk using a polyvinyl tube

**DOI:** 10.1007/s11748-022-01790-x

**Published:** 2022-03-04

**Authors:** Yusuke Inaba, Sachito Minegishi, Hidehito Endo, Hiroshi Kubota

**Affiliations:** Department of Cardiovascular Surgery, Kyorin University, 6-20-2 Shinkawa, Mitaka City, Tokyo Japan

**Keywords:** Frozen elephant trunk, Polyvinyl tube, Total arch replacement

## Abstract

The increasing use of total arch replacement and frozen elephant trunk (FET) is expected to lead to an increase in the prevalence of graft infections requiring replacement involving an FET, which is generally a more invasive procedure than the initial surgery. Herein, we describe a novel method for FET removal using a polyvinyl tube as the storage device. The procedure is completed using the same median sternotomy approach as the initial surgery. We report on the outcomes for six patients. Our procedure is simple to perform and safe and caused no injury to the aorta in our case series.

**Supplementary Information:**

The online version contains supplementary material available at 10.1007/s11748-022-01790-x.

## Introduction

Over the last decade, the frozen elephant trunk (FET) technique has replaced the conventional elephant trunk procedure for the treatment of complex and extensive aortic arch aneurysms. However, graft infection is a concern. Deep sternal wound infection (DSWI) is a rare but potentially devastating complication of the median sternotomy that is performed during cardiac surgery, with an incidence rate of 0.2–3% [1]. In contrast, the incidence of thoracic aortic graft infection is as high as 6%, with an associated mortality rate of up to 75%, depending on the clinical presentation [2]. As the use of total arch replacement (TAR) using FET continues to increase, the prevalence of graft infections involving an FET is also likely to increase. The treatment of FET graft infection requires the removal of all infected graft material, debridement of the surrounding tissue, and in situ reconstruction of the aortic arch with a new graft [3]. To replace the aorta in the area of the previous FET, a surgical approach with an additional incision line performed via a clam shell incision or a left thoracotomy is typically used. However, this approach carries inherent risk because of pre-existing infection and the generally poor health status of the patient. Moreover, removal of the FET, which is generally expanded and deployed within the tortuous distal aorta arch, is technically challenging. To address these issues, we have developed a novel approach for complete FET removal using a polyvinyl tube as the storage device via a median sternotomy; this technique is less invasive and can be completed within the same surgical field as the initial surgery. Herein, we describe our method for complete FET removal and the outcomes of six patients.

## Technique

**Fig. 1:**
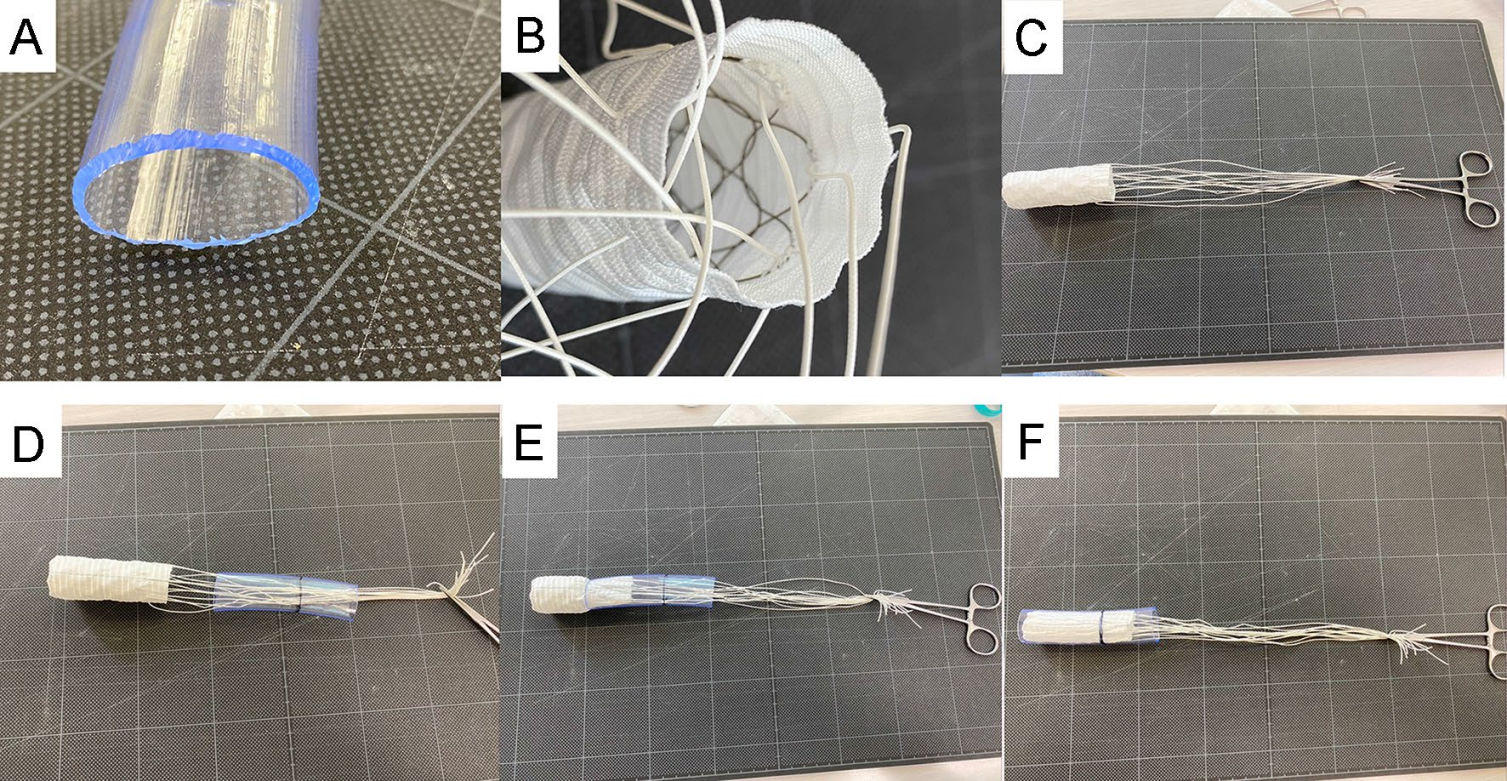
**a** Rounded and shaved distal tip of the polyvinyl tube. **b**–**c** No. 2 silk thread hanging from the stent. **d** No. 2 thread passing through the polyvinyl tube. **e** FET delivery into the polyvinyl tube for removal. **f** The FET is completely enclosed within the polyvinyl tube. *FET* frozen elephant trunk

**Fig. 2:**
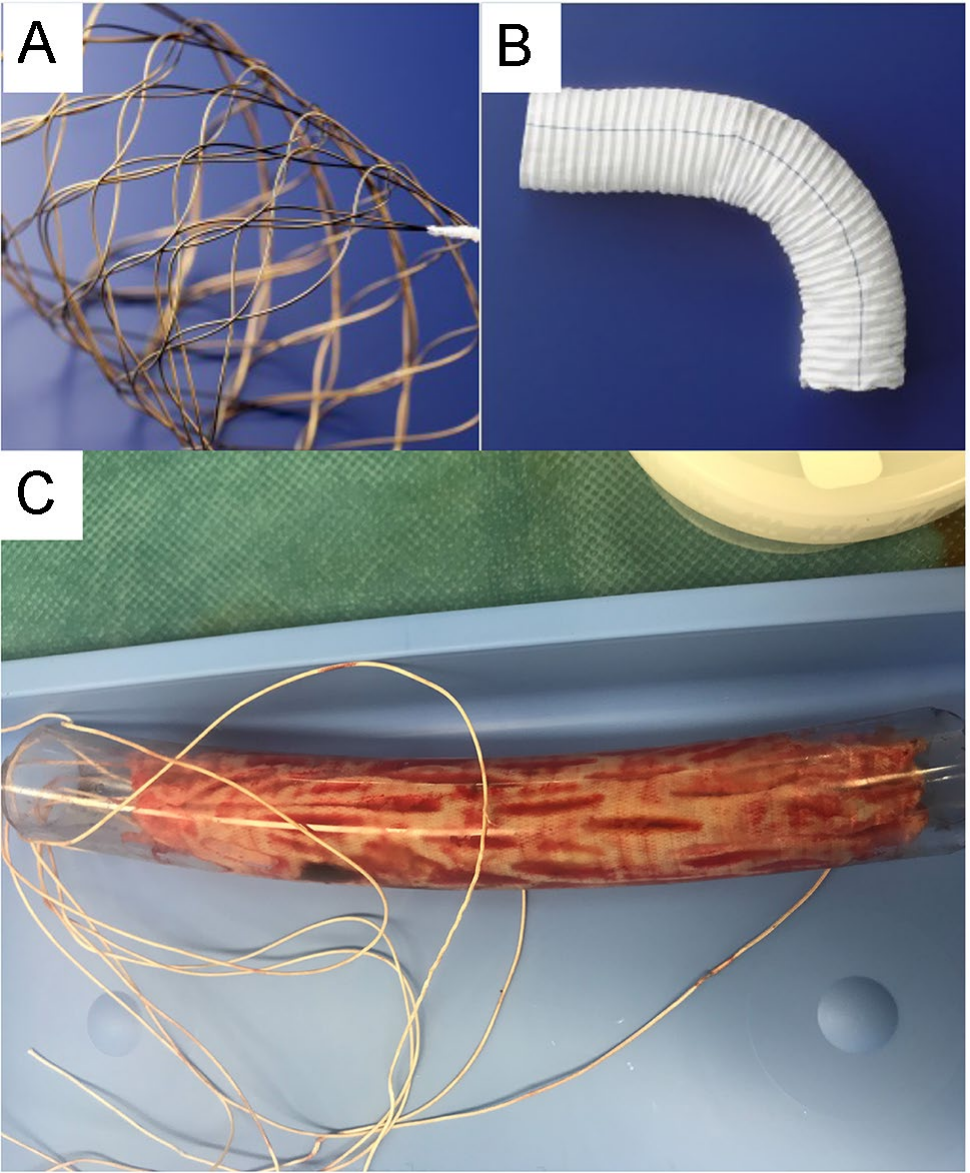
**a** Stent skeleton of the J-graft Frozenix. **b** External view of the J-graft Frozenix. **c** The Zenith Alpha thoracic endovascular stent graft is completely enclosed within the polyvinyl tube and then removed. **d** The J-graft Frozenix (25 × 120 mm) is completely enclosed within the polyvinyl tube and then removed. See Movie 1 of the procedure

Our approach to removing the FET from the distal arch to the descending aorta uses a polyvinyl tube as a storage device and is performed via a median sternotomy. We use a commercially available polyvinyl water supply tube (inner diameter: 19 mm, outer diameter: 23 mm) cut to a length 2 cm longer than the distal end-point of the graft to be removed. After a rounded end is formed to avoid damaging the aortic intima (Fig. 1a), the tube is sterilized using ethylene oxide gas. An external marking on the tube is used as an external indicator of the length to be inserted to reach the distal end-point of the stent graft to be removed, as it is not possible to gage this distance by observation once the tube is inserted into the aorta. A hook with a No. 2 silk thread is placed on the proximal stent end of the FET graft (Fig. 1b–c); the silk thread is passed through the polyvinyl tube (Fig. 1d) and tension is applied to pull the FET into the polyvinyl tube (Fig. 1e), with the polyvinyl tube sliding between the aorta and the FET. The resistance to insert gradually decreases as the FET graft is stored to the polyvinyl tube. Once the leading edge of the FET has reached the target reference line, the FET is completely stored within the tube and can be removed smoothly. After removing the FET, the absence of damage to the aortic intima is confirmed by endoscopy (Fig. 1f; 2d; Video 1). A new anastomosis site on the aorta is trimmed, and TAR is performed. In all six reported cases, FET removal was indicated eliminate all foreign material because all four branched graft was surrounded by abscess cavity from deep sternal wound infection and blood culture was positive for all patients. FET removal was indicated because of four branched graft infections from DSWI. The primary indication for TAR and FET in all the cases was a Stanford type A acute aortic dissection, and a J-graft Frozenix® (Japan LifeLine, Tokyo, Japan) had been used. The period from the initial surgery to FET removal was 42 ± 16 days (range, 14–58 days), and FETs of different dimensions: 29 × 120 mm (*n* = 2), 27 × 120 mm (*n* = 1), 27 × 90 mm (*n* = 1), 27 × 60 mm (*n* = 1), and 25 × 90 mm (*n* = 1) were removed in all cases with poly vinyl tubes of the same size. Culture findings were positive in three of the six removed FET grafts. All FET could be removed without difficulty regardless the duration between the primary operation and FET removal. No adverse events associated with our method were noted during the postoperative period.

## Comments

The FET technique, first described by Kato et al. [4], consists of the insertion of a self-made stent graft into the distal arch, performed under cardiopulmonary bypass, to treat an extended aortic aneurysm or dissection. The indications for TAR and FET have been expanded to include emergent cases in addition to elective cases [5]. Various FETs are commercially available. The J-graft Frozenix FET consists of a Dacron polyester fabric vascular prosthesis with a nitinol stent inside the graft (Fig. 2a–b).

Our method has the advantage of using the same operative field as the initial surgery, which is less invasive and can lower the risk of infection. The use of a 20-cc syringe or proctoscope, as previously described for the treatment of endovascular aneurysm repair (EVAR) infection [6], is impractical, since these devices cannot follow the shape of the aortic arch and their use can result in damage to the aortic intima. The polyvinyl tube used in our technique is sufficiently flexible to follow the shape of the aortic arch, while having sufficient hardness to prevent kinking as it is pushed through the aorta. The following tips can improve the ease and safety of our procedure: avoid placing the silk thread under excessive tension, fixing the FET using an anchor, and sliding the tube between the aorta and the FET. We used a No. 2 silk thread to pull the FET into the tube, as a silk thread of lesser diameter could not withstand the tension developed during the procedure. We noted that tension from the thread is applied only to the stent component of the FET, which protects against tearing of the graft.

We have also used our method to remove an infected Zenith Alpha thoracic endovascular graft (Cook Medical, Bloomington, Indiana, USA) deployed from the infected prosthetics graft to the native descending aorta. For the Zenith Alpha, a No. 2 silk thread was passed through the uncovered stent, allowing for complete storage of the stent graft within the polyvinyl tube. Therefore, our technique is effective for the removal of both the J-graft Frozenix, in which the stent is located inside the prosthetic graft, and the Zenith Alpha, in which the stent is located outside the graft (Fig. 2c). We believe that our technique might be suitable for the removal all types of FET and thoracic endovascular aortic repair stents. Our procedure is not indicated when there were obvious findings of the infection in the native aorta at the FET insertion site.

In summary, we report a novel technique for the removal of FET using a polyvinyl tube as a storage device. The FET was successfully removed in all six reported cases without damaging the intima of the aortic wall. Therefore, we consider our technique to be simple and safe. However, careful observation of the anastomosis is necessary. To our knowledge, this is the first report describing FET removal using a storage device. The further development of techniques and medical products for FET removal is warranted for further advances in the field of thoracic aortic surgery.

## Supplementary Information

Below is the link to the electronic supplementary material.

Supplementary file1 (MP4 68785 KB)
